# A Third Generation Glucose Biosensor Based on Cellobiose Dehydrogenase Immobilized on a Glassy Carbon Electrode Decorated with Electrodeposited Gold Nanoparticles: Characterization and Application in Human Saliva

**DOI:** 10.3390/s17081912

**Published:** 2017-08-18

**Authors:** Paolo Bollella, Lo Gorton, Roland Ludwig, Riccarda Antiochia

**Affiliations:** 1 Department of Chemistry and Drug Technologies, Sapienza University of Rome, P.le Aldo Moro. 5, 00185 Rome, Italy; paolo.bollella@uniroma1.it; 2 Department of Analytical Chemistry/Biochemistry and Structural Biology, Lund University, P.O. Box 124, SE-221 00 Lund, Sweden; lo.gorton@biochemistry.lu.se; 3 Food Biotechnology Laboratory, Department of Food Science and Technology, BOKU—University of Natural Resources and Life Sciences, Muthgasse 18, A-1190 Vienna, Austria; roland.ludwig@boku.ac.at

**Keywords:** cellobiose dehydrogenase, gold nanoparticles, electrodeposition, glucose biosensor, human saliva

## Abstract

Efficient direct electron transfer (DET) between a cellobiose dehydrogenase mutant from *Corynascus thermophilus* (*Ct*CDH C291Y) and a novel glassy carbon (GC)-modified electrode, obtained by direct electrodeposition of gold nanoparticles (AuNPs) was realized. The electrode was further modified with a mixed self-assembled monolayer of 4-aminothiophenol (4-APh) and 4-mercaptobenzoic acid (4-MBA), by using glutaraldehyde (GA) as cross-linking agent. The *Ct*CDH C291Y/GA/4-APh,4-MBA/AuNPs/GC platform showed an apparent heterogeneous electron transfer rate constant (*k*_s_) of 19.4 ± 0.6 s^−1^, with an enhanced theoretical and real enzyme surface coverage (*Γ*_theor_ and *Γ*_real_) of 5287 ± 152 pmol cm^−2^ and 27 ± 2 pmol cm^−2^, respectively. The modified electrode was successively used as glucose biosensor exhibiting a detection limit of 6.2 μM, an extended linear range from 0.02 to 30 mM, a sensitivity of 3.1 ± 0.1 μA mM^−1^ cm^−2^ (R^2^ = 0.995), excellent stability and good selectivity. These performances compared favourably with other glucose biosensors reported in the literature. Finally, the biosensor was tested to quantify the glucose content in human saliva samples with successful results in terms of both recovery and correlation with glucose blood levels, allowing further considerations on the development of non-invasive glucose monitoring devices.

## 1. Introduction

Glucose monitoring has attracted great attention in several fields, ranging from biomedical applications to ecological fields [1]. In particular, for clinical trials, glucose monitoring has been considered one of the key factor in early diagnosis of diabetes mellitus, which is a main cause of death or other diseases around the world. Diabetes is a metabolic disease generally related to non-/under-production of insulin in the pancreas and hyperglycemia, reflected by blood glucose concentrations higher or lower than the normal range of 80–120 mg dL^−1^ [2]. It is possible to distinguish between three types of diabetes: (i) type 1, which most affects young people, with non-production of insulin in the pancreas and involves about 10% of diabetic people [3]; (ii) type 2, which occurs in middle-age or old people, with low production of insulin or when the body does not use the insulin produced and involves about 90% of diabetic people [4]; (iii) gestational diabetes, which occurs during the pregnancy, with a connected risk of diabetes development for both mother and child [5].

In the last century, several approaches for early diagnosis of diabetes mellitus mainly focused on glucose monitoring have been developed such as capillary zone electrophoresis (CZE) [6], gas chromatography (GC) mainly coupled with mass spectrometry (MS) [7], high performance liquid chromatography/mass spectrometry (HPLC-MS) [8], enzymatic spectrophotometric assays [9], Fourier transform near-infrared spectroscopy (FT-NIR) [10] and proton NMR.

However, these methods require expensive equipment and complicated operations and therefore they cannot be applied to home-based care [11]. Most diabetic patients need to test their blood glucose levels periodically, even several times a day. Biosensors may represent a valid alternative as they allow time-saving, accurate, repeatable and cost-effective determination of glucose in blood [12]. Typically, a blood test for analysis is realized through a finger prick, which may cause physical and mental stress to patients, especially to young children and elderly people. Therefore, there is a great need for the developing of a point-of-care non-invasive glucose monitoring system [2]. A positive correlation between blood glucose (80–120 mg dL^−1^) and salivary glucose (0.6–1.8 mg dL^−1^) has been revealed in many studies [4]. 

Saliva shows great advantages compared to other biological fluids such as blood, tears, urine, etc. because its sampling is not invasive and it is recognized as the most sensitive one, containing also other disease biomarkers in a concentration larger than in blood. Moreover saliva sampling involves a simple collection method that allows easy storage and transport. 

It is well known that electrochemical enzymatic glucose biosensors are divided into three classes depending on the electrochemical communication between the enzyme and the electrode: (i) first generation biosensors, where glucose is considered as co-substrate of glucose oxidase (GOx) with a subsequent generation of hydrogen peroxide (H_2_O_2_), which is oxidised at the electrode surface at quite high redox potential (e.g., 0.6 V vs. Ag|AgCl_sat_ for carbon modified electrodes, 0.4 V Ag|AgCl_sat_ for gold electrodes and 0.7 V vs. Ag|AgCl_sat_ for platinum electrode; (ii) second generation biosensors, where oxygen was replaced with a non-physiological electron acceptor (mediator) able to shuttle the electrons from the enzyme redox center to the surface of the electrode; (iii) third generation biosensors, where the mediator was eliminated to develop a reagentless glucose biosensor transferring the electrons from glucose to the electrode through the active site of the enzyme, with a low operating potential, close to that of the redox potential of the enzyme itself [13]. The electrode nanostructure plays a key role, particularly for third generation biosensors, thanks to the increased electroactive area and roughness factor [13]. Among the various nanostructured materials, carbon nanotubes, graphene [14] and metal nanoparticles (MNPs) showed very promising results [15], because of their high surface/volume ratio, thus allowing a better communication between the electrode and the prosthetic group of the enzyme [16]. Recently, several MNPs deposition approaches have been developed to modify the electrode surface, such as drop-casting [17,18], covalent linkage [19,20] or direct electrodeposition [21]. Among these methods, the electrodeposition allows a fast and easy MNPs synthesis with the possibility to monitor the MNPs geometry and size [22], especially by sweeping the potential [23] instead of applying a fixed potential [24]. Moreover, MNPs can grow directly onto the electrode surface without the need of further sample preparation, being surfactant-free and cost-effective and allowing to tune the nature of the nanoclusters by changing electrolyte composition and deposition parameters [24].

Unfortunately, despite the electrode nanostructure, only a few enzymes are able to directly transfer electrons from their active sites to the electrode [25,26]. Cellobiose dehydrogenase (CDH) has received great attention for biosensors [27] and biofuel cell development [28] because of its ability to show DET. CDH is a flavocytochrome oxidoreductase expressed by the dikaryotic phyla of Basidiomycota (Class I) and Ascomycota (Class II and Class III), consisting of two domains [29]. The first domain, called dehydrogenase domain (DH_CDH_), contains a flavin adenine dinucleotide (FAD) cofactor, connected through a flexible linker to a second subunit containing a *heme-b* cofactor, called cytochrome domain (CYT_CDH_) [30]. DH_CDH_ domain is structurally similar to the FAD domain of most GMC-oxidoreductase enzymes and is fully reduced by di-/mono- saccharides, transferring the electrons through internal electron transfer (IET) to the CYT_CDH_, which finally shuttles the electrons to properly modified electrodes [31]. Among II class CDHs, *Corynascus thermophilus* CDH (*Ct*CDH) was genetically mutated in its active site (*Ct*CDH C291Y mutant) to enhance its sensitivity toward glucose and reduce the maltose cross-reactivity [32]. 

**Scheme 1:**
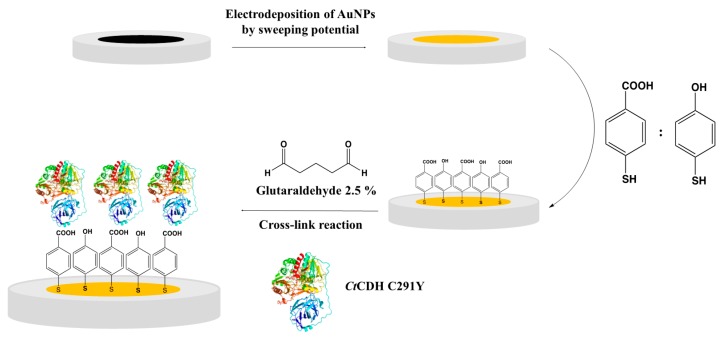
The electrode modification pathway for *Ct*CDH C291Y/GA/4-APh,4-MBA/AuNPs/GC platform has been reported. Initially, AuNPs were directly electrodeposited onto cleaned GC electrode by sweeping the potential. Afterward, the electrode was incubated in a thiol mixture (1:1 *v*/*v* 4-APh and 4-MBA) overnight, followed by cross-linking reaction (glutaraldehyde GA: cross-linking agent) to covalently link the enzyme (*Ct*CDH C291Y) to the so modified electrode surface.

In this work, we report an improved DET efficiency between *Ct*CDH C291Y and a novel GC modified electrode, obtained through direct electrodeposition of gold nanoparticles (AuNPs) on the GC electrode, further modified with a mixed self-assembled monolayer of 4-aminothiophenol (4-APh) and 4-mercaptobenzoic acid (4-MBA) using glutaraldehyde as cross-linking agent, as shown in Scheme 1. The proposed electrodeposition method allowed to monitor the nanoparticles surface coverage as well as the surface area available for the biomodification, which is directly related to the biosensor sensitivity. The so modified AuNPs/GC electrode was used to develop a third generation biosensor for glucose detection. The performances of the proposed biosensor were investigated in human saliva samples, demonstrating that the constructed AuNPs/GC biosensor has great potentials to realize electrochemical devices for non-invasive diabetes mellitus monitoring.

## 2. Experimental Section

### 2.1. Chemicals

Sulfuric acid (H_2_SO_4_), d-glucose, d-(+)-maltose monohydrate, ascorbic acid, calcium chloride (CaCl_2_), chloroauric acid (HAuCl_4_·3H_2_O), potassium ferricyanide (K_3_[Fe(CN)_6_]), potassium ferrocyanide (K_4_[Fe(CN)_6_]), sodium acetate (CH_3_COOH), 3-(*N*-morpholino)propanesulfonic acid (MOPS), tris(hydroxymethyl)aminomethane (TRIS), urea, cortisol, 4-aminothiophenol (4-APh), 4-mercaptobenzoic acid (4-MBA), glutaraldehyde (GA), potassium chloride (KCl) and Glucose (GO) Assay Kit were purchased from Sigma Aldrich (St. Louis, MO, USA). 

*Ct*CDH C291Y (E.C. 1.1.99.18) was purified from the culture supernatant of the ascomycete *Corynascus thermophilus* (CBS 405.69) obtained from the Centralbureau voor Schimmelcultures (Baarn, The Netherlands) (volumetric activity with cytochrome *c* at pH 7.5 = 54 U mL^−1^, protein concentration = 16 mg mL^−1^). All solutions were prepared using Milli-Q water (R = 18.2 MΩ cm at 25 °C; TOC < 10 μg L^−1^, Millipore, Molsheim, France).

### 2.2. Electrode Preparation and Modification

GC electrodes (Bioanalytical Systems Inc., West Lafayette, IN, USA, d = 3 mm) were polished with alumina slurries (Al_2_O_3_, particle size of 1 and 0.1 μm) on cloth pads wet with Milli-Q water (Struers ApS, Ballerup, Denmark), thoroughly rinsed with Milli-Q water and further sonicated for 5 min between each polishing step. GC electrodes were successively modified by electrodeposition of gold nanoparticles (AuNPs) by sweeping the potential between 1.1 and −0.1 V vs. Ag|AgCl_sat_ for a given number of scans (5, 10, 15, 20, 25, 30, 35 scans) in 10 mM HAuCl_4_ [33]. Then, the modified electrodes were activated in 0.5 M H_2_SO_4_ by running 25 scans between 0 and +1.7 vs. Ag|AgCl_sat_ at a scan rate of 0.1 V s^−1^ until a well-defined cyclic voltammogram (CV) was obtained. The best modified electrode was selected on the basis of the electroactive and real surface area, heterogeneous electron transfer rate constant (*k*^0^, cm s^−1^) and roughness factor (*ρ*), calculated from CV measurements carried out in 10 mM Fe(CN)_6_^3−/4−^ (50 mM TRIS buffer pH 7.4). It was further dipped into a volumetric 1:1 mixture of 1 mM 4-APh/4-MBA ethanol solution. Then, the electrode was thoroughly rinsed with ethanol and dried under N_2_ stream. For the biomodification, 1 μL of GA solution (2.5% *v*/*v* in distilled water) and 3 μL of *Ct*CDH C291Y solution (16 mg mL^−1^) were drop-cast, gently mixed on the top of thiol-modified AuNPs/GC electrode and allowed to react in a moisturised atmosphere for 2 h to avoid evaporation of the reactants. Finally, the so modified electrode was gently rinsed with 50 mM TRIS buffer (pH 7.4) in order to remove any possible unbounded enzyme molecule [34]. 

### 2.3. Whole Saliva and Blood Samples Collection and Analysis

Saliva samples collection was performed at 8.30 a.m. from three healthy male and female patients refrained from eating, drinking and oral hygiene procedures (at least for 1 h before). The patients were given drinking bottled water and asked to rinse well their mouths. After 5 min, the patients were asked to spit whole saliva (WS) into a 50 mL sterile Falcon^®^ tube, once a minute for up to 10 min until sampling 5 mL of WS [35]. At the same time the patients were punched on their fingers to collect a drop of blood sufficient to measure glucose with the commercial GlucoContour XT (Bayer, Leverkusen, Germany) used by diabetic patients for self-monitoring and with the glucose oxidase-peroxidase method [36,37] by using the Glucose (GO) Assay Kit, which is the standard reference method for WS samples [38].

### 2.4. SEM Experiments

Scanned electron microscopy (SEM) measurements were performed with a JSM-7600F Schottky Field Emission Scanning Electron Microscope (JEOL Nordic AB, Sollentuna, Sweden). All samples were prepared according to the electrodeposition protocol, reported in Section 2.2, using glassy carbon plates (25 × 25 × 1 mm, ALS Co. Ltd., Tokyo, Japan) instead of GC electrodes. The samples were paced on a clip SEM sample holder (JEOL Nordic AB).

### 2.5. Electrochemical Measurements and Electrochemical Apparatus

Cyclic voltammograms (CVs) were recorded by using a PGSTAT 30 potentiostat (equipped with GPES 4.9, Autolab, Utrecht, The Netherlands). CVs were performed in a three-electrode electrochemical cell containing a standard silver chloride electrode (Ag|AgCl, sat. KCl), a platinum wire counter electrode and a modified glassy carbon (GC) electrode as working electrode. The temperature controlled experiments were carried out by using a cryostatic bath (T ± 0.01 °C, LAUDA RM6, Delran, NJ, USA). Flow injection analysis (FIA) data have been collected by using an analogic potentiostat (Zäta Elektronik, Höör, Sweden) connected with a strip chart recorder (Kipp & Zonen, Utrecht, The Netherlands). The modified GC electrode, an Ag|AgCl (0.1 M KCl) reference electrode and a platinum wire counter electrode were fitted into a wall-jet cell. The electrochemical system was equipped with a flow system consisting of a peristaltic pump (Gilson, Villier-le-Bel, France) and a six-port valve electrical injector (Rheodyne, Cotati, CA, USA).

## 3. Results and Discussion

### 3.1. SEM and Electrochemical Characterization of AuNPs Modified GC Electrodes 

**Figure 1:**
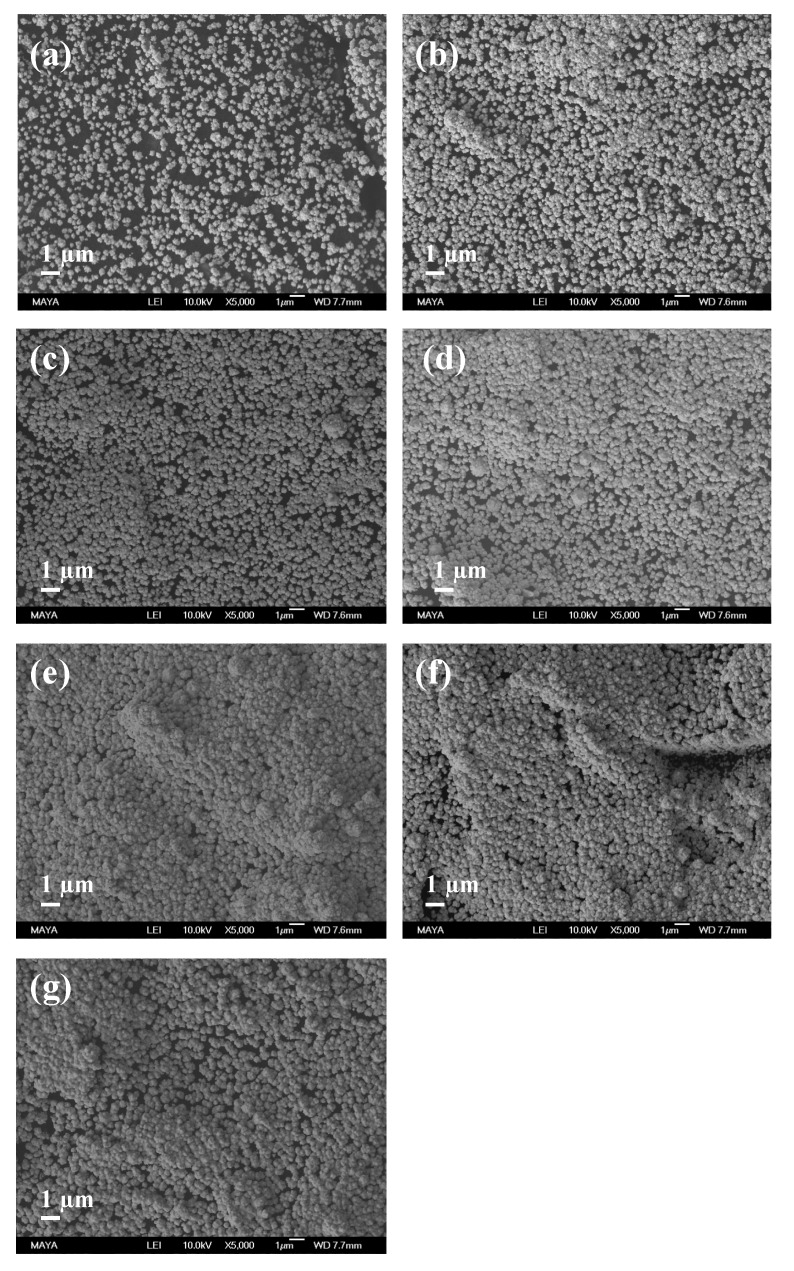
SEM images of AuNPs-modified GCEs obtained by sweeping the potential between 1.1 and −0.1 V vs. Ag|AgCl_sat_ for a given number of scans in 10 mM HAuCl_4_ (0.5 M H_2_SO_4_): (**a**) 5, (**b**) 10, (**c**) 15, (**d**) 20, (**e**) 25, (**f**) 30 and (**g**) 35.

SEMs were used to evaluate the physical appearance and surface characteristics of the AuNPs on the electrode surfaces for a given number of scans. Figure 1a–g show the SEM images relative to increasing number of scans. It is clearly visible that the surface coverage of the AuNPs increases with increasing number of scans until 25 scans, when the electrode surface is completely covered by a single layer of AuNPs. For electrodes prepared with 30 and 35 scans (Figure 1f,g), it is possible to observe multiple layers of AuNPs with possible formation of AuNPs agglomerates. 

**Figure 2:**
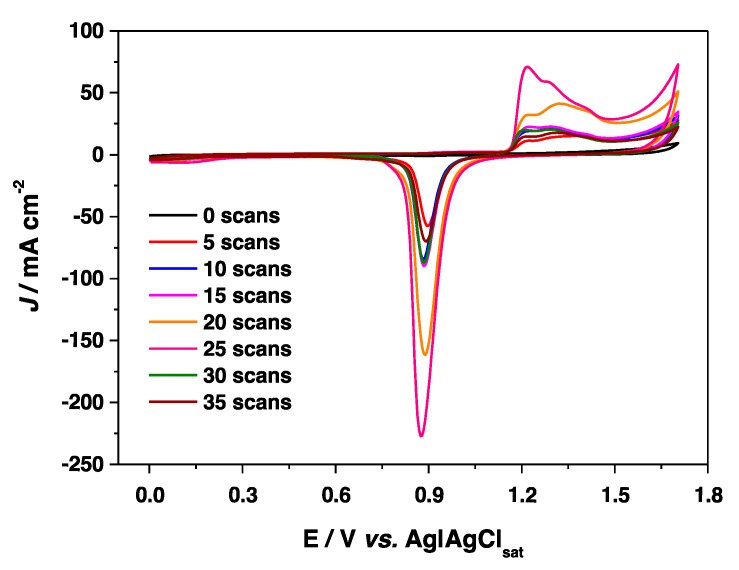
Cyclic voltammograms of AuNPs modified GCE electrodes with different number of scans (from 0 scans to 35 scans) in 0.5 M H_2_SO_4_. Scan rate 100 mV s^−1^. T = 25 °C.

All AuNP-modified electrodes were successively characterized by cyclic voltammetry (CV) experiments in a solution of Fe(CN)_6_^3−/4−^ (data not shown) in order to calculate the electroactive area (*A_EA_*, cm^2^), the heterogeneous electron transfer rate constant (*k*^0^, cm s^−1^) and the roughness factor (electroactive/geometrical area ratio, *ρ*) and in 0.5 M H_2_SO_4_ (Figure 2) in order to calculate the real surface area (*A_real_*). All data are shown in Table 1. The A_EA_ has been evaluated using the Randles-Sevcik equation by the slope of the peak current vs. square root of scan rate ($\upsilon^{1/2}$) [39], whereas the real surface area (*A_real_*) was calculated by integration of the peak current related to the gold oxide reduction process occurring by running CVs in 0.5 M H_2_SO_4_ [40,41]. The theoretical charge density considered for gold oxide reduction is 390 ± 10 μC cm^−2^ [42]. *k*^0^ was calculated using the extended method which merges the Klingler-Kochi and Nicholson-Shain methods for totally irreversible and reversible systems, respectively [43,44]. It is possible to observe in Table 1 that all the electrochemical parameters are highly influenced by the number of scans, showing the best results after 25 scans with an A_EA_ of 12.96 ± 0.18 cm^2^ and a roughness factor of 183.6 ± 1.2, probably related to the increase in AuNPs surface coverage with the scan number. With electrodes prepared with 30 and 35 scans the decrease in the electrochemical parameters reported in Table 1 might be due to the presence of multiple layers and possible AuNPs agglomeration (Figure 1f,g). 

### 3.2. Electrochemistry of CtCDH C291Y on Modified GA/4-APh,4-MBA/AuNPs/GC Electrode

**Figure 3:**
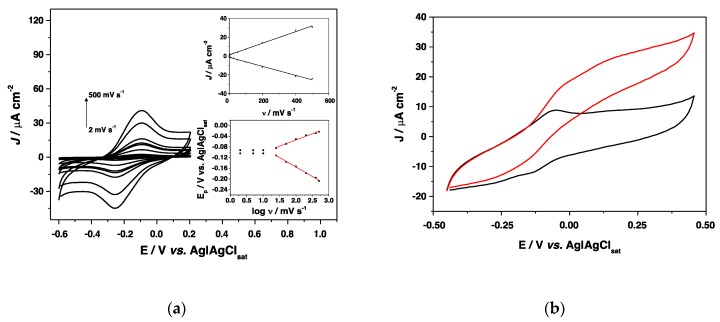
(**a**) CVs of *Ct*CDH C291Y/GA/4-APh,4-MBA/AuNPs/GC in pH 7.4, 50 mM TRIS (0.1 M KCl) in the potential range of −0.6 V to 0.35 V, scan rates from 2 mV s^−1^ to 500 mV s^−1^. Inset: linear part of cathodic (*J*_pc_) and anodic peak current densities (*J*_pa_) vs. scan rate (*v*). (**b**) CVs of *Ct*CDH C291Y/GA/4-APh, 4-MBA/AuNPs/GC in absence (black) and in presence of 5 mM glucose (red) in 50 mM TRIS buffer, pH 7.4 (0.1 M KCl) at a scan rate of 5 mV s^−1^. T = 25 °C.

After preliminary characterization, the modified AuNPs/GC electrode obtained after 25 scans of electrodeposition was further modified with *Ct*CDH C291Y covalently linked through GA with a mixed SAM consisting of 4-APh and 4-MBA. Figure 3a depicts the typical CVs of the enzyme electrode at different scan rates, showing an increased peak-to-peak separation (*ΔE_p_*) between the anodic and cathodic peak potentials. The modified electrode exhibited a clear linear dependence of both anodic and cathodic peak current densities versus the scan rate over the range 2–500 mV s^−1^, as shown in the inset of Figure 3a. The presented results fitted with thin-layer electrochemical behaviour, as generally reported for immobilized systems.

It is possible to observe in Figure 3b (black curve) a couple of peaks related to DET of CDH through the CYT_CDH_ subunit containing the *heme b*, which displayed a midpoint potential (*E^0^’*) of −98 mV vs. Ag|AgCl_sat_, close to the values reported in the literature for Ascomycota CDHs immobilized on gold electrodes [19]. The apparent heterogeneous electron transfer rate constant (*k*_s_) was calculated by considering an electron transfer coefficient of 0.53, obtained by fitting the linear part of the trumpet plot, as shown in the inset of Figure 3a. Therefore, the *k*_s_ value was estimated to be 19.4 ± 0.6 s^−1^, according to Laviron’s equation [45] reported below:

(1) $$\log k_{s}=\;\alpha \log\left(1-\alpha \right)+\left(1-\alpha \right)\log\left(RT/nF\upsilon \right)-\alpha \left(1-\alpha \right)nF\Delta E_{p}/2.3RT$$

where *α* is the electron transfer coefficient, *n* the number of electrons, *ΔE_p_* the separation of the redox peak potentials and *ν* the scan rate (*F* = 96.495 C mol^−1^, *T* = 298 K, *R* = 8.31 J mol^−1^ K^−1^).

By integration of the redox peaks relative to the DET of CYT_CDH_ it was possible to evaluate the enzyme surface coverage using the Faraday’s law below reported in Equation (2):

(2) $$\Gamma_{T}=\frac{Q}{nFA}$$

where *Γ_T_* is the total surface concentration of electroactive protein (mol cm^−2^), *A* the electrode area (cm^2^), *F* the Faraday’s constant (96 495 C mol^−1^ of electrons), *Q* the charge underlying the redox wave and *n* the number of electrons [46]. The theoretical surface coverage (*Γ*_theor_) was estimated to be 5287 ± 152 pmol cm^−2^ (*A_geom_* = 0.073 cm^2^), while the real surface coverage (*Γ*_real_) resulted to be 27 ± 2 pmol cm^−2^ (*A_real_* = 13.83 ± 0.04 cm^2^, as shown in Table 1). Afterwards, the electrocatalytic behaviour of the *Ct*CDH C291Y/GA/4-APh,4-MBA/AuNPs/GC electrode was studied by performing CVs in the presence of 5 mM glucose as substrate (Figure 3b, red curve), showing excellent performances with a current density of about 30 μA cm^−2^, probably due to the high nanostructuration of the electrode surface and the covalent immobilization of the enzyme.

### 3.3. Glucose Biosensor Development

The amperometric response to glucose was studied by injecting glucose solutions at different concentrations by using the flow injection analysis (FIA) system, in order to investigate the electroanalytical and kinetic parameters of the modified *Ct*CDH C291Y/GA/4-APh,4-MBA/AuNPs/GC electrode. The biosensor showed a fast peak response (5 s), probably due to the enlarged surface area related to the electrodeposition of the AuNPs and the cross-linking of the enzyme, which ensure high number of immobilized enzyme molecules and stable enzyme layer. 

**Figure 4:**
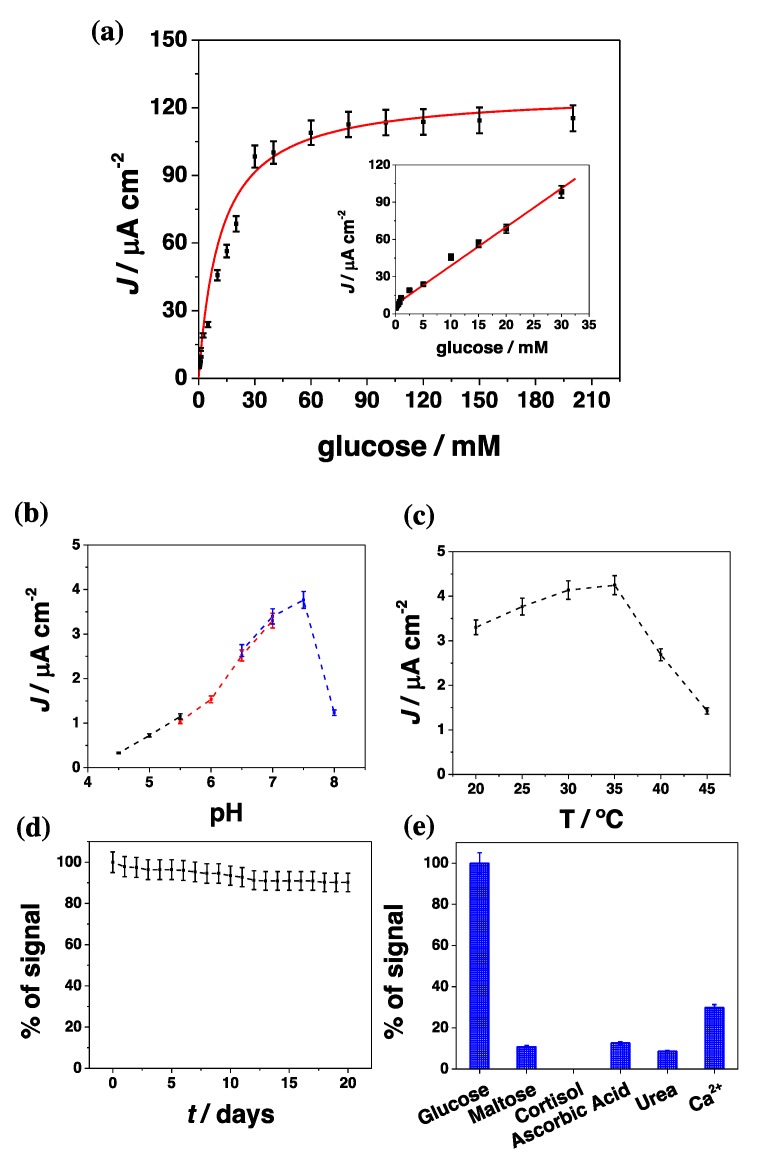
(**a**) Glucose biosensor calibration graph of *Ct*CDH C291Y/GA/4-APh,4-MBA/AuNPs/GC in 50 TRIS buffer pH 7.4 (0.1 M KCl); *E_app_* = 0.250 vs. Ag|AgCl_sat_; flow rate 0.5 mL min^−1^; inset: linear part of the calibration curve; (**b**) biosensor response over the pH range (4.5–8): 50 mM acetate buffer (black), 50 mM MOPS buffer (red) and in 50 mM TRIS buffer (blue), with *E_app_* = 0.250 vs. Ag|AgCl_sat_; flow rate 0.5 mL min^−1^; (**c**) biosensor response over the T range (20–45 °C) in TRIS buffer; (**d**) lifetime of *Ct*CDH C291Y/GA/4-APh,4-MBA/AuNPs/GC biosensor in presence of 750 μM glucose solution; (**e**) influence of interfering compounds on glucose response in presence of 750 μM glucose, maltose, cortisol, ascorbic acid, Ca^2+^. Experimental conditions (**c**–**e**): 50 TRIS buffer pH 7.4 (0.1 M KCl), *E_app_* = 0.250 vs. Ag|AgCl_sat_; flow rate 0.5 mL min^−1^; injection volume 50 μL; T = 25 °C.

The calibration curve displayed a linear response range between 0.02 and 30 mM (R^2^ = 0.995, *n* = 5) with a sensitivity of 3.1 ± 0.1 μA mM^−1^ cm^−2^, as shown in the inset of Figure 4a. At higher concentrations the amperometric response is no longer linear due to the saturation of the enzyme active site. The detection limit for *Ct*CDH C291Y/GA/4-APh,4-MBA/AuNPs/GC biosensor was found to be 6.2 μM, calculated using the relation 3σ/S, where σ is the absolute standard deviation of the intercept and S is the slope of the calibration curve [47]. The analytical performances of the glucose biosensor and the kinetic parameters are listed in Table 2. The apparent kinetic parameters (*I*_max_, *K_M_^app^*) are in good agreement with the values reported in the literature for nanostructured electrodes [48]. It is interesting to underline that today very few third generation glucose biosensors based on Ascomycota CDHs have been reported in the literature while most other glucose biosensors are based on first and second generation electron transfer mechanism of other GMC oxidoreductase enzymes (e.g., GOx).

The proposed *Ct*CDH C291Y/GA/4-APh,4-MBA/AuNPs/GC biosensor shows a clear increase in terms of sensitivity, selectivity, stability, extended linear range and lower detection limit compared to other second and third generation glucose biosensors reported in the literature, as shown in Table 3. 

Nevertheless, it exhibits a lower sensitivity compared to the first generation glucose biosensors, probably because of some issues related to the DET [49,50,51,52,53,54,55]. On the other hand, first and second generation biosensors have known drawbacks, such as their instability and the toxicity of the mediator layer.

### 3.4. Effect of pH and Temperature, Interferences and Stability Studies

The effects of pH and temperature on the proposed glucose biosensor were evaluated and the results are reported in Figure 4b. The optimum pH resulted to be pH 7 in TRIS buffer at a temperature of 35 °C. A significant decrease in the current densities occurs below pH 5.5 and above 8, in perfect agreement with the data reported in the literature about the optimum pH of the free CDH [56]. The dependence on the temperature is shown in Figure 4c where it is possible to see that the amperometric response increased from 20 to 30–35 °C and drastically decreased above 37 °C, due to a possible inactivation of the enzyme caused by the temperature.

The stability and lifetime of the *Ct*CDH C291Y/GA/4-APh,4-MBA/AuNPs/GC biosensor was evaluated using the FIA system by monitoring the signal decrease within 20 days when the biosensor is used for one measurement per day, as reported in Figure 4d. The modified biosensor seems to retain about 90% of its initial activity after 20 days, probably due to the stability of the enzyme layer directly related to the nanostructuration of the electrode surface.

Finally, the selectivity of the proposed biosensor was studied in order to see the influence of possible interfering compounds generally present in human saliva such as maltose, cortisol, ascorbic acid, urea and calcium ions (Ca^2+^). The signal obtained for a fixed concentration of glucose (750 μM) was compared to that obtained with a sample containing the same glucose concentration plus equal amounts of the possible interfering compounds. The amperometric signal is lower than 10% for all compounds tested with the exception of Ca^2+^ ions, which potentially may interfere in real measurements (30% of glucose signal), probably because of its interaction with some amino acid residues present between the DH_CDH_ and CYT_CDH_ domains [56].

### 3.5. Glucose Detection in Human Saliva 

In order to demonstrate the feasibility of the modified electrode for the non-invasive detection of glucose, the proposed biosensor was used to detect the concentration of glucose in human saliva samples. The samples were collected according to the procedure reported in Section 2.3, referred to literature on saliva analysis. The reliability of the amperometric biosensor platform *Ct*CDH C291Y/GA/4-APh,4-MBA/AuNPs/GC was evaluated by comparing the results with those obtained with the glucose oxidase-peroxidase method. The proposed biosensor showed satisfactory results in all samples tested with a recovery between 95.0 and 97.4% (RSD values lower than 4%), as reported in Table 4. 

**Figure 5:**
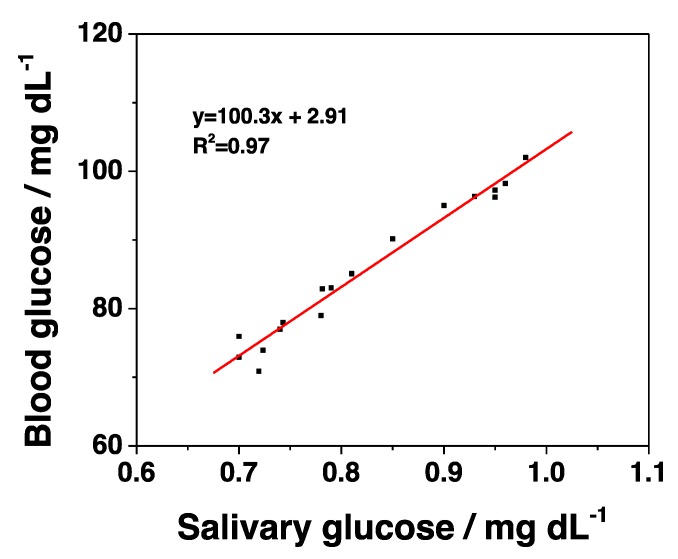
Correlation between glucose blood and saliva glucose concentration in 6 healthy patients at fasting state. Salivary glucose was detected with the *Ct*CDH C291Y/GA/4-APh,4-MBA/AuNPs/GC biosensor, blood glucose was detected with the commercial GlucoContour XT.

The glucose content was measured also in blood samples collected from the same healthy patients with a commercial self-monitoring system (GlucoContour XT) in order to evaluate the correlation between glucose saliva and blood levels, for future potential development of devices for non-invasive glucose monitoring [57]. Figure 5 shows a good correlation between salivary and blood glucose concentration, opening the doors to the development of possible self-non invasive glucose monitoring devices.

## 4. Conclusions

We have demonstrated the possibility to carefully monitor the surface coverage of AuNPs on the electrode surface through a direct electrochemical deposition method of AuNPs onto a glassy carbon electrode which allows to achieve an efficient DET thanks to the effective nanostructure and the cross-linking of CDH molecules. AuNPs resulted to be very efficient for retaining the enzyme activity and promoting the electron transfer. The *Ct*CDH C291Y/GA/4-APh,4-MBA/AuNPs/GC biosensor showed great performances in terms of extended linear range and higher sensitivity, selectivity and stability compared to other glucose biosensors. The promising platform allowed the detection of glucose in human saliva with results in very good agreement with those obtained with the standard spectrophotometric method showing also a good correlation with glucose blood levels. For these reasons, the proposed biosensor may represent the basis for the development of a portable non-invasive device for glucose monitoring in diabetes mellitus patients. 

## Figures and Tables

**Table 1:** Characterization of AuNPs on GC modified electrodes for different numbers of cyclic scans (N) during the electrodeposition step. Experimental conditions: 10 mM Fe(CN)_6_^3−/4−^ in 50 mM TRIS buffer pH 7.4 with 0.1 M KCl for electroactive area (*A_EA_*), real surface area (*A_real_*), electron transfer rate constant (*k*^0^) and roughness factor (*ρ*) determination; 0.5 M H_2_SO_4_ at a scan rate of 100 mV s^−1^, for *A_real_* determination.

Scan Numbers (N)	*A_EA_*/cm^2^	*A_real_*/cm^2^	*k^0^_app_*/10^−2^ cm s^−1^	Roughness Factor (*ρ*)
0	0.09 ± 0.01	-	4.6 ± 0.1	1.3 ± 0.1
5	2.72 ± 0.21	2.93 ± 0.07	5.2 ± 0.3	38.5 ± 0.3
10	3.92 ± 0.12	4.13 ± 0.06	7.9 ± 0.4	55.5 ± 0.3
15	4.13 ± 0.19	4.46 ± 0.06	13.4 ± 0.6	58.5 ± 0.5
20	8.74 ± 0.25	9.26 ± 0.04	18.2 ± 0.7	123.8 ± 0.8
25	12.96 ± 0.18	13.83 ± 0.04	26.8 ± 0.3	183.6 ± 1.2
30	4.18 ± 0.06	4.39 ± 0.08	15.3 ± 0.8	59.2 ± 0.7
35	3.26 ± 0.12	3.99 ± 0.06	10.6 ± 0.5	46.2 ± 1.3

**Table 2:** Electroanalytical and kinetic parameters of the *Ct*CDH C291Y/GA/4-APh,4-MBA/AuNPs/GC biosensor obtained by FIA amperometry in 50 mM TRIS buffer, pH 7.4. Applied potential: +0.250 V vs. Ag|AgCl_sat_.

	*Ct*CDH C291Y/GA/4-APh,4-MBA/AuNPs/GC
E_app_/(V vs. Ag|AgCl_sat_)	+0.250
K_M_^app^/mM	11.7 ± 1.2
*J*_max_/μA cm^−2^	126.9 ± 3.0
LOD/μM	6.2
Linear range/mM	0.02–30
Sensitivity/μA mM^−1^ cm^−2^	3.1 ± 0.1
R^2^	0.99

**Table 3:** Comparison between glucose biosensors based on mediated (MET) and direct electron transfer (DET). List of abbreviations: 1,1′-dimethylferrocene (DMFc), 1,2-diaminobenzene (p-DAB), 4-aminophenol (4-APh), 4-mercaptobenzoic acid (4-MBA), *Corynascus thermophilus* cellobiose dehydrogenase (*Ct*CDH), *Corynascus thermophilus* cellobiose dehydrogenase C291Y mutant (*Ct*CDH C291Y), ferrocene-COOH (Fc-COOH), glassy carbon electrode (GC), glucose oxidase (GOx), glutaraldehyde (GA), gold (Au), gold nanoparticles (AuNPs), osmium (bpy)_2_poly (1-vinylimidazole) (PVI-Os), Prussian Blue (PB), screen-printed carbon electrode (SPCE), single walled carbon nanotubes (SWCNTs), titanium nanotubes (TiNTs).

Modified Electrode	*E_app_*/V vs. Ag|AgCl_sat_	Linear Range/mM	LOD/μM	Sensitivity/μA mM^−1^ cm^−2^	Stability	Reference
(GOx/Au/pDAB)-PB/AuNPs/TiNTs (1^st^ generation)	−0.35	0.01–0.70	3.2	248.0	>90% after 1 month	[49]
GOx-GA/PB/Pt (1^st^ generation)	−0.05	0.01–1	5	80.0	83.3% after 23 days	[50]
GOx/PB/SPCE (1^st^ generation)	−0.30	0.25–2	10.0	17.0	72% after 45 days	[51]
GOx/Fc-COOH/SPCE (2^nd^ generation)	+0.25	1–5	180.0	2.0	~80% after 3 weeks	[52]
GOx/DMFc/G (2^nd^ generation)	+0.16	1–30	300	-	-	[53]
GOx-SWCNTs-PVI-Os/SPCE (2^nd^ generation)	+0.30	0.2–7.5	0.07	32	~90% after 30 days	[54]
*Ct*CDH/GA/SWCNTs/SPCE (3^rd^ generation)	+0.10	0.1–30	10.0	-	~90% after 7.5 h	[55]
*Ct*CDH C291Y/GA/4-APh, 4-MBA/AuNPs/GC (3^rd^ generation)	+0.25	0.02–30	6.2	3.1	~90% after 20 days	this work

**Table 4:** Glucose measurements in human saliva with the *Ct*CDH C291Y/GA/4-APh,4-MBA/AuNPs/GC biosensor and with the glucose oxidase-peroxidase kit (GlucoContour XT), as reference method. Experimental conditions: 50 mM TRIS buffer pH 7.4, *E_app_* = +0.250 V vs. Ag|AgCl_sat_.

	Biosensor/mg dL^−1^	Reference Method/mg dL^−1^	Recovery/%
Sample 1 (male)	0.77 ± 0.03	0.79 ± 0.03	97.4
Sample 2 (male)	0.96 ± 0.02	1.00 ± 0.04	95.7
Sample 3 (male)	0.89 ± 0.03	0.92 ± 0.05	96.9
Sample 1 (female)	0.74 ± 0.03	0.75 ± 0.03	98.8
Sample 2 (female)	0.71 ± 0.01	0.74 ± 0.02	95.0
Sample 3 (female)	0.77 ± 0.02	0.80 ± 0.01	96.5
